# Whether Letrozole could reduce the incidence of early ovary hyperstimulation syndrome after assisted reproductive technology? A systematic review and meta-analysis

**DOI:** 10.1186/s12978-020-01042-2

**Published:** 2020-11-20

**Authors:** Jing Zhao, Bin Xu, Xi Huang, Yi Yan, Yanping Li

**Affiliations:** Reproductive Medicine Center, Xiangya Hospital, Central South University, 87 Xiangya Road, Changsha, Hunan People’s Republic of China

**Keywords:** Ovarian hyperstimulation syndrome, Letrozole, Assisted reproductive technologies

## Abstract

**Background:**

Letrozole can significantly decrease the estrogen level, and has been administrated to prevent the incidence of early ovary hyperstimulation syndrome (OHSS). However, the effect of Letrozole on prevention of OHSS reached to controversial conclusions. The present meta-analysis aim to examine whether Letrozole could reduce the incidence of early OHSS after assisted reproductive technology (ART).

**Methods:**

An exhaustive electronic literature search was conducted on MEDLINE, Google Scholar, CNKI and WANFANG MED ONLINE, from inception until May 2018. We include clinical trials that examined the effect of Letrozole on the prevention of early OHSS. The main outcome measures were the incidence of total early OHSS, mild early OHSS, moderate early OHSS, and severe early OHSS.

**Results:**

Eight studies included in the review. Of these, five publications evaluated the effect of Letrozolel on the prevention of total, mild, moderate, and severe OHSS, respectively. The results indicated that there was a significantly decreased incidence of total OHSS with Letrozole compared with control group, and there were no significantly differences in the incidence of mild, moderate, and severe OHSS between study group with Letrozole and control group. Eight studies reported the incidence of moderate + severe OHSS. We found a significant decrease in incidence of moderate + severe OHSS in high-risk women with Letrozole.

**Conclusions:**

Letrozole has no beneficial effect on the prevention of mild, moderate, and severe OHSS, individually; Letrozole should not be considered as the first-line treatment for prevention of OHSS. Further cohort studies are required to explore the effect of Letrozole on the prevention of OHSS.

**Plain English Summary:**

This study aimed to examine whether Letrozole could reduce the incidence of early OHSS after assisted reproductive technology (ART). A meta-analysis including 8 studies was conducted. There were no significantly differences in the incidence of mild, moderate, and severe OHSS between study group with Letrozole and control group. Letrozole has no beneficial effect on the prevention of mild, moderate, and severe OHSS, individually.

## Background

Ovarian hyperstimulation syndrome (OHSS) is a serious iatrogenic complication resulted by controlled ovarian stimulation (COS) during assisted reproductive technology (ART). It was reported that 3.1–8.0% of in vitro fertilization (IVF) cycles developed into moderate to severe OHSS, and the incidence rate has reached to 20% in women at high-risk [1]. The clinical manifestation of OHSS includes enlarged ovarian volume, abdominal tenderness and swelling, which caused by an increased vascular permeability and effusion to the extravascular space. Lyons et al. [2] described 2 forms of OHSS for the first time: the early and late forms. The early OHSS happened within 9 days after HCG trigger and the late OHSS occurs 10 days after HCG trigger and is always associated with endogenous HCG level after pregnancy.

Vascular endothelial growth factor (VEGF) plays a crucial part in the development of OHSS [3, 4]. Besides, other systematic and local vasoactive substances are also directly and indirectly involved in the pathogenesis of OHSS symptoms [3, 5–9]. At present, there have no effective methods that can treat moderate and severe OHSS, so prevention seems very important [10]. For the women at high-risk of OHSS, an important means to prevent OHSS is to cryopreserve all fresh embryos [11, 12]. However, only late OHSS can be avoided by fresh embryo cryopreservation, but early OHSS cannot be prevented [13]. A number of strategies have been suggested to reduce the incidence of OHSS, such as coasting [14], GnRH-ant protocol with GnRH-a for trigger [15, 16], HES [17, 18], human albumin [19], aspirin [20, 21], dopamine agonist [22], calcium [23], metformin [24], aspiration of pleural effusion [25].

As we all known, elevated serum estrogen concentrations have been correlated with a higher incidence of OHSS [26]. Letrozole is a nonsteroidal aromatase inhibitor, and can inhibit the conversion of androgens into estrogens by blocking the aromatase and E synthetase in a potent, specific, and reversible way [27]. One study showed that administrating Letrozole after oocyte retrieval could reduce E2 level and restore LH production, and proposed that Letrozole may be used to reduce the OHSS incidence [28, 29]. In the following years, several clinical trails have evaluated the effect of Letrozole on the prevention of early OHSS in high-risk women. Some studies have reported that Letrozole can reduce the incidence of early OHSS [30–34]. However, some studies have showed that Letrozole can only reduce the E2 level, but failed to prevent the occurrence of OHSS [35–37]. So whether the Letrozole should be given to prevent OHSS reached to controversial conclusions, which made both clinicians and infertile women in an awkward position.

In the present systematic review, we aim to further evaluate whether Letrozole have effect on the prevention of early OHSS during ART in high-risk women and perform a systematic review and meta-analysis of the available literatures.

## Materials and methods

### Identification of the literature

Electronic databases including MEDLINE, Google Scholar, CNKI and WANFANG MED ONLINE, were searched from inception until May 2018. The key words used to search relative studies were as follows: one including terms on Letrozole (“Letrozole”, “Aromatase inhibitor”), one including terms on OHSS (“ovulary hyperstimulation syndrome”, “OHSS”), and the last one about reproductive technologies (“IVF”, “ICSI”, “ART”, “in vitro fertilization”, “intracytoplasmic sperm injection”, “assisted reproductive technology”). A subset of citations related to the present question were generated by combining these subsets with “AND”. Two authors independently evaluated the eligibility of the articles, and group discussion was needed when there was a discrepancy.

### Study selection and data extraction

Studies that administered Letrozole to prevent the OHSS in high-risk women undergoing COS were selected. The primary outcome was the incidence of total OHSS, the incidence of mild, moderate and severe OHSS. For studies to be eligible, outcome data were extracted in 2 × 2 tables. We also recorded the study type, treatment of ART, the ovary stimulation protocol, and the inclusion criteria of women. The quality of the included studies was evaluated by two authors with the Newcastle–Ottawa Quality Assessment Scales [38], a third author is needed if there was any disagreements about inclusion.

### Statistical analysis

The comparisons of included studies were analyzed by a standard meta-analytic method, and the combined results were expressed with the odds relatives (ORs) and its 95% confidence interval (CI). Heterogeneity of the studies was evaluated graphically using Forest plot and statistically using the *I*^2^ to quantify heterogeneity between studies. A fixed effect model or a random effect model was implied to calculate an overall OR and its 95% CI. Statistical analyses were carried out with RevMan 5.0 (Cochrane Collaboration). The results were considered to be statistically significant when the P value was < 0.05..

## Results

### Studies selection and characteristics

**Fig. 1:**
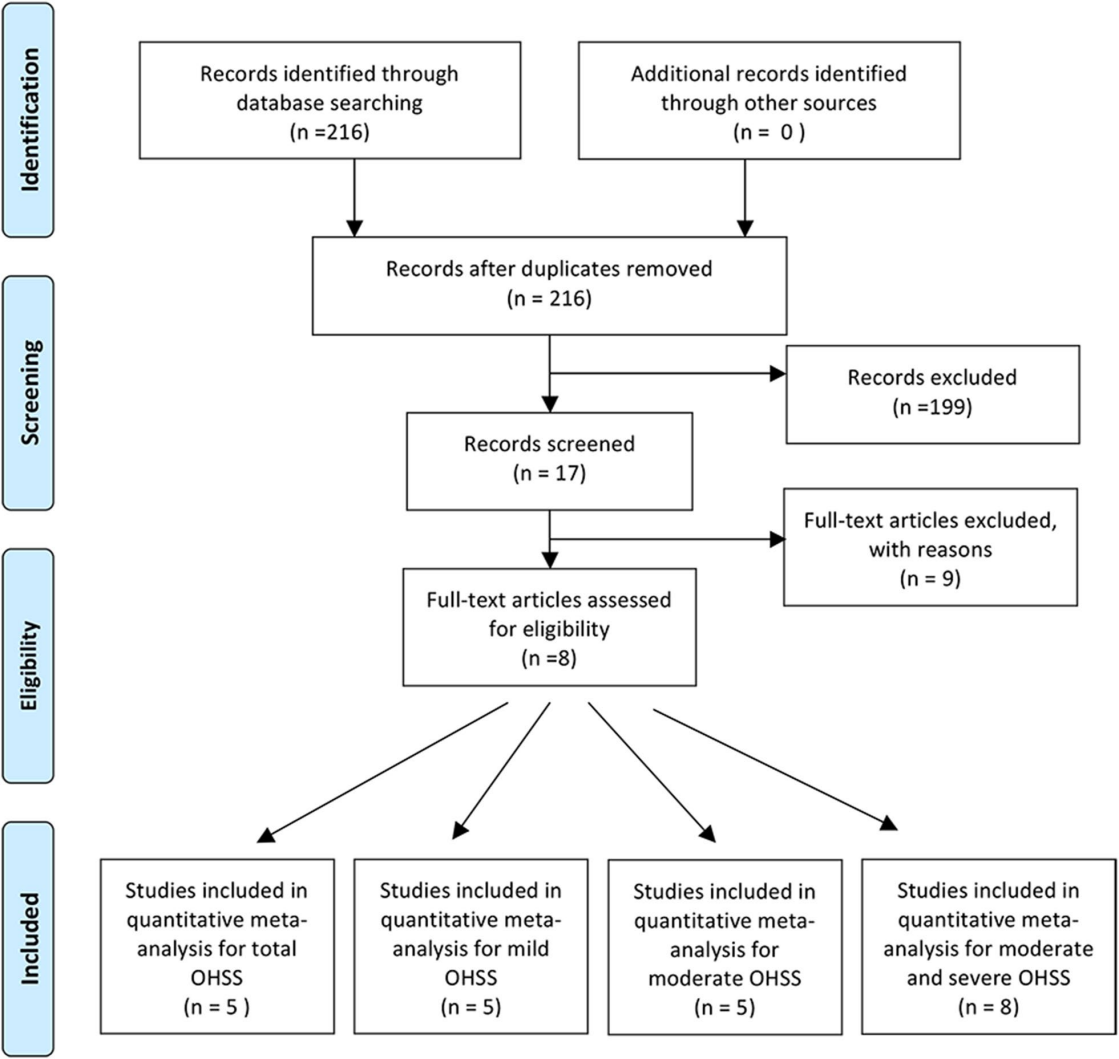
Flow chart showing study selection process

216 articles were yielded with the above search strategy. Of there, 199 were not relevant after reviewing the titles and abstracts. Of the 17 remaining studies, one were excluded because the incidence of OHSS was not reported; Two studies have no full-text available. Three studies were excluded because all their data were duplications of another three studies that have been included in our review; an additional three articles were excluded because of lack of a 2 × 2 table. (Fig. 1).

The total number of eligible studies included in the review was 8, comprising 1551 women. Of these, five publications including 1215 women evaluated the effect of Letrozole administration on the prevention of total OHSS and mild OHSS. Five studies including 770 women reported the incidence of moderate OHSS and severe OHSS. Eight studies reported the incidence of moderate + severe OHSS.

The studies’ characteristics are summarized in Table 1. Of these 8 articles, 6 were prospective studies, and 2 were retrospective. All the studies used long GnRH-a protocol. Four studies only used Letrozole in the study group, and other four studies used both Letrozole and other treatments at the same time. As for the control group, three studies received oral placebo or no medication, and other drugs were administered in control group of the other five studies. Six of these studies evaluated the severity of OHSS according to the study by Golan et al. [39], one study according to Navot’s study [40], and another one study did not mention it.

**Table 1:** Characteristics of studies included in the systematic review and meta-analysis of letrozole and OHSS after ART

Study	Type of study	Treatment	Protocol	Inclusion criteria	Usage of letrozole	Control	Standard for OHSS	Outcome
He et al. [32]	Prospective	IVF, ICSI	Standard long protocol	No. of oocyte retrieval ≥ 25 E2 ≥ 6000 pg/ml on the HCG day Ovary diameter ≥ 10 cm Puncture follicle No. ≥ 30	2.5 mg qd /2.5 mg bid/2.5 mg tid, for 5 days	Oral placebo for 5 days	Golan et al	Moderate and severe
Wang et al. [36]	Prospective	IVF	Long mid-luteal GnRH-a protocol	No. of oocyte retrieval ≥ 20 No. of follicles greater than 14 mm ≥ 20 E2 ≥ 8000 pg/ml on the HCG day Ovary diameter ≥ 10 cm	2.5 mg p.o. Bid for 5 days	Receive no special medication	Golan et al	Mild, moderate, severe
Mai et al. [30]	Prospective	IVF,ICSI,PGD	Midluteal long GnRH-a protocol/short GnRH-a suppressive protocol	Oocyte retrieval ≥ 25 Estradiol level ≥ 5000 pg/ml Clinical or ultrasonographic evidence of OHSS	2.5 mg p.o. Bid for 5 days	Aspirin 100 mg qd, for 5 days	Navot D, et al	Mild, moderate, severe
Wang et al. [35]	Prospective	IVF	Long mid-luteal GnRH-a protocol	No. of oocyte retrieval ≥ 25 No. of follicles greater than 14 mm ≥ 25 E2 ≥ 8000 pg/ml on the HCG day Ovary diameter ≥ 10 cm	Letrozole 2.5 mg p.o. Bid for 5 days + support therapy	Receive support therapy alone	Golan et al	Mild, moderate, severe
Lin et al. [37]	Retrospective	IVF, ICSI	Long mid-luteal GnRH-a protocol	E2 ≥ 7000 pg/ml on the HCG day 7000 pg/ml > E2 ≥ 5000 pg/ml, and No. oocytes retrieval is 15 ~ 20, and abdominal distension No. of oocyte retrieval ≥ 20	Letrozole 2.5 mg p.o. Bid for 5 days + HES + prednisone 5 mg tid	Hydroxyethyl starch(HES) + prednisone 5 mg tid	No mention	Moderate and severe
Yu [33]	Prospective	IVF, ICSI	Long mid-luteal GnRH-a protocol	No. of oocyte retrieval > 20 E2 > 17765 pmol/l No. of follicles greater than 14 mm > 20 Ovary diameter ≥ 10 cm Other severe clinical mannifestations	Letrozole + HES + Ca	HES + Calcium gluconate (Ca)	Golan et al	Mild, Moderate, severe
Zhang et al. [34]	Retrospective	IVF,ICSI	GnRH-a long protocol/ultra-long GnRH-a protocol	No. of oocyte retrieval ≥ 20 E2 ≥ 25620 pmol/L Obvious abdominal distension	Letrozole 2.5 mg p.o. tid for 10 days + HES	HES	Golan et al	Total OHSS, moderate and severe
He et al. [31]	Prospective	IVF, ICSI	Standard long protocol	No. of oocyte retrieval ≥ 25 E2 ≥ 22020 pmol/l No. of follicles greater than 14 mm ≥ 30 Ovary diameter ≥ 10 cm	Letrozole 2.5 g bid, for 5 days	Placebo bid, for 5 days	Golan et al	Moderate, severe

### Meta-analysis

**Fig. 2:**
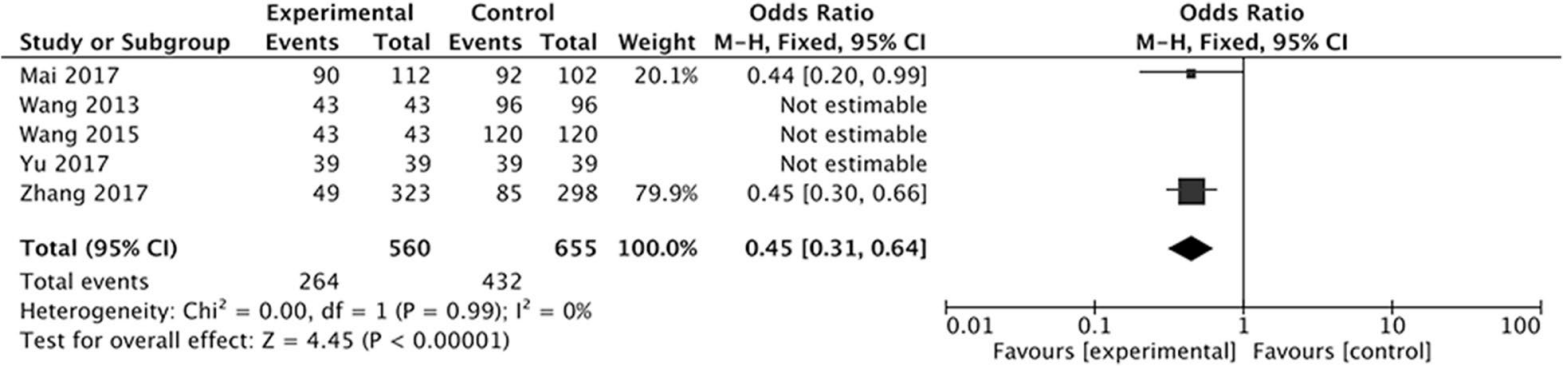
Forest plot showing the results of meta-analysis of studies evaluating the effect of letrozole administration on prevention of total early OHSS after ART

At first, we evaluated the effect of Letrozole administration on the prevention of total OHSS after ART. Five studies were included in our meta-analysis. The result indicated a significantly decreased incidence of total OHSS with administration of Letrozole compared with control group (OR 0.45; 95% CI 0.31, 0.64; *P* < 0.00001), and there was good statistical heterogeneity in the results (*I*^2^ = 0%, *P* = 0.99) (Fig. 2).

**Fig. 3:**
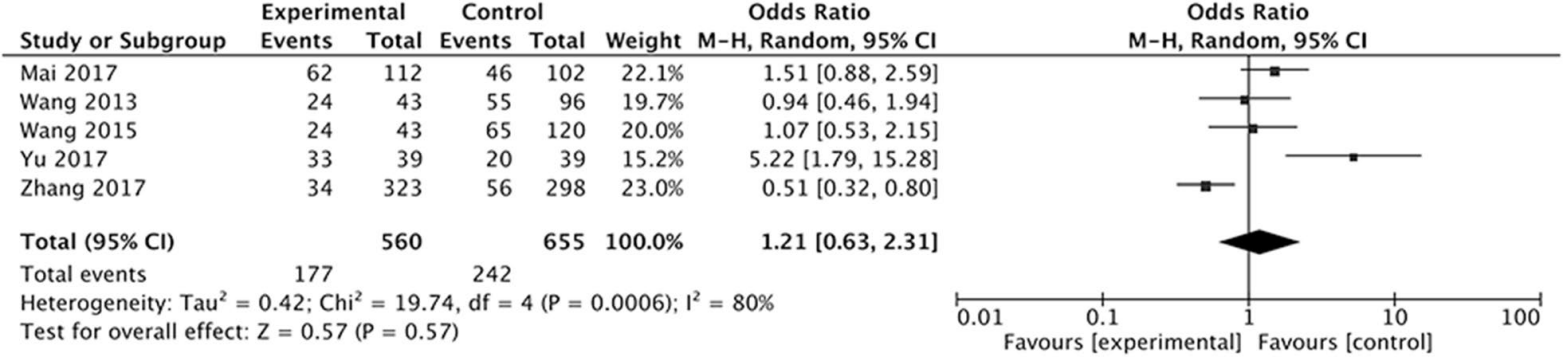
Forest plot showing the results of meta-analysis of studies evaluating the effect of letrozole administration on prevention of mild early OHSS after ART

The above five studies also evaluated the effect of Letrozole administration on the prevention of mild OHSS after ART at the same time. After analysis, we found that there was no significantly difference in the incidence of mild OHSS between study group with Letrozole administration and control group with no medication or other treatment. The Q statistic P value was < 0.05, indicating heterogeneity of the studies (I^2^ = 80%, P = 0.0006). The random effects model was used and the combined OR was 1.21 (95% CI 0.63, 2.31; P = 0.57) (Fig. 3).

**Fig. 4:**
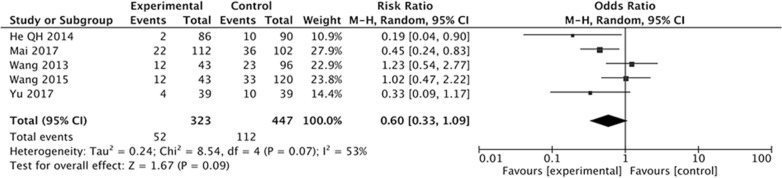
Forest plot showing the results of meta-analysis of studies evaluating the effect of letrozole administration on prevention of moderate early OHSS after ART

The effect Letrozole administration on the prevention of moderate OHSS was also evaluated. Five studies with 770 women were included outcomes. The results indicated that there was no significantly difference in incidence of moderate OHSS between study group with Letrozole administration and control group with no medication or other treatments (OR 0.60; 95% CI 0.33, 1.09; P = 0.09) (Fig. 4). The studies evaluating the incidence of moderate OHSS showed moderate heterogeneity (*I*^2^ = 53%, *P* = 0.07).

**Fig. 5:**
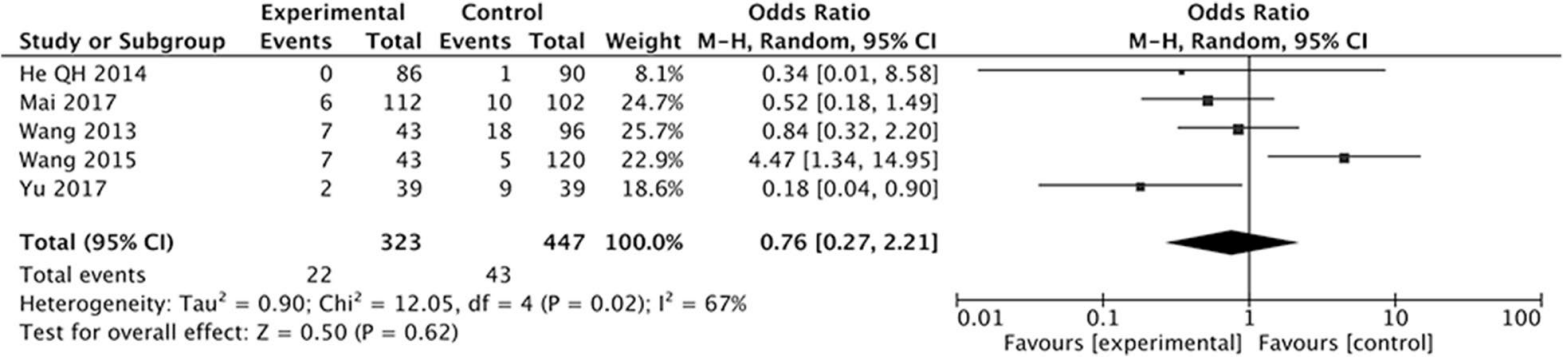
Forest plot showing the results of meta-analysis of studies evaluating the effect of letrozole administration on prevention of severe early OHSS after ART

When we evaluated the effect of Letrozole on the prevention of severe OHSS after ART in high-risk women, five studies were included. The result of the meta-analysis showed that there was similar incidence of severe OHSS between study group with Letrozole administration and the control group with or without other treatments. There exist heterogeneity of the studies (*I*^2^ = 67%, *P* = 0.02), as the *Q* statistic *P*-value was below 0.05. We implied the random effects model and the combined OR was 0.76 (95% CI 0.27, 2.21; *P* = 0.62) (Fig. 5).

**Fig. 6:**
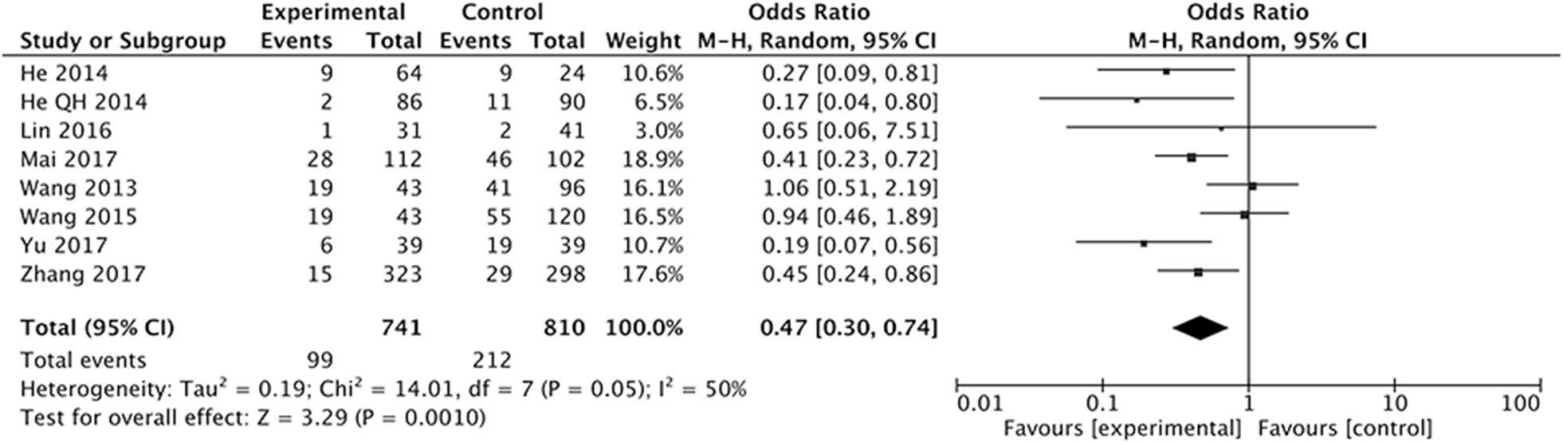
Forest plot showing the results of meta-analysis of studies evaluating the effect of letrozole administration on prevention of moderate + severe early OHSS after ART

Eight studies were included to assess the effect of Letrozole on the prevention of moderate + severe OHSS after ART. We found a significant decreased incidence of moderate + severe OHSS in high-risk women with Letrozole compared with those with or without other medication in the control group. The *Q* statistic *P* value was 0.05, showing heterogeneity of the studies (*I*^2^ = 50%, P = 0.05). The random effects model combined odds ratio (OR) was 0.47 (95% CI 0.30–0.74; P = 0.001) (Fig. 6).

The studies have got good marks with the Newcastle–Ottawa Quality Assessment Scale (not shown). The funnel plots evaluating the effect of Letrozole on the prevention of total OHSS, mild, severe, and moderate + severe OHSS suggest lack of publication bias, thanks to their symmetrical shape (Additional file 1: Figure S1, Additional file 2: Figure S2, Additional file 3: Figure S3, Additional file 4: Figure S4). However, the study indicated modest publication bias when assessing the effect of Letrozole on the prevention of moderate OHSS (Additional file 5: Figure S5).

## Discussion

To the best of our knowledge, the present study is the first systematic review and meta-analysis, which assess the effect of Letrozole on the prevention of early OHSS after ART. Many studies have explored the effect of Letrozole administration on prevention of OHSS after ovary stimulation during IVF/ICIS/PGD treatment cycles. Several studies found that Letrozole administration after oocyte retrieval could decrease the incidence of OHSS [30–34], while other studies did not show a decreased incidence of OHSS after ART with Letrozole administration [35–37]. In the present review and meta-analysis, 5, 5, 5, 5 and 8 studies were included to assess the effect of Letrozole administration on the prevention of total OHSS, mild OHSS, moderate OHSS, severe OHSS, and moderate-severe OHSS, respectively.

The results have demonstrated that the Letrozole administration could significantly decrease the incidence of total OHSS, the pooled OR was 0.45 (95% CI 0.31, 0.64). When we classify the OHSS into mild, moderate, and severe OHSS according to the severity, the results indicated that all kinds of OHSS seems cannot be prevented by Letrozole administration, the pooled ORs was 1.21 (95% CI 0.63, 2.31) for mild OHSS, 0.60 (95% CI 0.33, 1.09) for moderate OHSS, 0.76 (95% CI 0.27, 2.21). The incidence of moderate and severe OHSS showed a decreased tendency, but there were no significantly difference between study group with Letrozole administration and control group with no medication or other treatments.

In the clinical practice, there is no clear boundary between the moderate OHSS and the severe OHSS, and the moderate OHSS would develop into severe OHSS in a short time. So we in further evaluate whether Letrozole administration could decrease the incidence of the moderate + severe OHSS. Eight studies with 1551 women were included. The pooled OR was 0.47 with 95% CI 0.30, 0.74, indicating beneficial effect of Letrozole administration on prevention of combined moderate + severe OHSS. The difference between combined and individual analysis may be attribute to the sample size.

The characteristic of OHSS is multi-follicular development, enlarged ovarian, and high E_2_ [41, 42]. The serum E_2_ level on the day of HCG injection is an important indicator to predict the occurrence of OHSS [43]. When the level of E_2_ is more than 6000 pg/ml, the incidence rate of severe OHSS can up to 38% [44]. During COS, “coasting” program (Gn is stopped for several days) was used to reduce the level of E_2_ to safe concentration, and the HCG was administrated to induce oocyte final maturation, leading to decreased incidence of OHSS in the women at a high-risk [45]. This indicated that the serum E_2_ level is strongly related with the incidence of OHSS.

As a non-steroidal aromatase inhibitor, Letrozole can block the human aromatase, and inhibit androgens from converting to estrogens, thereby decreasing the E_2_ level [46]. At first, Letrozole was used in patients with E_2_-dependent tumors, such as breast cancer, and was used to induce ovulation [47]. In recent years, Letrozole was gradually applied in women with high risk OHSS. It is well known that the Letrozole can decrease the E_2_ level, but it is conflict whether Letrozole can decrease the incidence of OHSS.

In 2008, Fatemi reported that E2 level was significantly decreased after 5.0 mg Letrozole administration during luteal phase compared with placebo group [29]. In 2009, Garcia-Velasco reported that 2.5 mg of Letrozole can significantly decreased E2 during luteal phase, which was consistent with the pharmacological mechanism of Letrozole, and proposed that it could be used to prevent the OHSS [28].

Recently, some studies have tried Letrozole to prevent the occurrence of early OHSS. One study explored the effect of different doses of Letrozole on the incidence of OHSS after oocyte retrieval during IVF in patients with high-risk OHSS, and showed that 2.5 mg, 5.0 mg, and 7.5 mg daily for 5 days can decrease the level of E_2_ and VEGF to some extent in patients at high-risk for OHSS. The incidence of OHSS was slightly decreased with 2.5 mg and 5 mg Letrozole, and was significantly decreased with 7.5 mg Letrozole, indicating that 7.5 mg Letrozole may be useful to limit OHSS [31]. In 2017, another study by Mai compared the effect of Letrozole with aspirin in prevention of early OHSS, and found that Letrozole was more effective than aspirin in decreasing the incidence of moderate and severe early-onset OHSS. In this study, author indicated that OHSS might be caused by a luteolytic effect rather modulation of VEGF [30].

However, Wang described that 5 mg of Letrozole during luteal phase can significantly decrease serum E2 levels on the 2^nd^, 5^th^, and 8^th^ days after oocyte retrieval compared with the control group, but it could not decrease the incidence of severe OHSS [35]. Two years later, her team explored the effect of ovarian steroid hormone suppression during luteal phase after oocyte retrieval on prevention of severe OHSS in high-risk women with all fresh embryo cryopreservation, and showed that steroidal ovarian suppression with Letrozole or mifepristone or cetrotide seems not to be able to prevent severe OHSS [36].

At present, the guideline for “Prevention and Treatment of moderate and severe ovarian hyperstimulation syndrome” has not commended Letrozole to prevent OHSS [48]. So it is very necessary to do the present systematic review and meta-analysis. Interestingly, the present study showed that Letrozole could decrease the incidence of total OHSS and moderate + severe OHSS, whereas have no effect on the prevention of occurrence of mild, moderate, and severe OHSS, individually. This is consistent with results reported by Wang et al. [35, 36].

The possible explanations for our results were as follows: Firstly, the sample size was relative small when three kinds of OHSS were evaluated individually. Secondly, Letrozole have no effect on prevention of OHSS. As Wang have state, high E2 level could only predict the occurrence of OHSS, and is not the reason for OHSS, so decreased E2 level during luteal phase could not prevent the incidence of OHSS. Thirdly, exuberant secretion from multiple corpus luteum after superovulation can cause the high E2 levels observed during the luteal phase. From the point of pathogenesis and pathophysiology, aromatase inhibitor administration during the luteal phase cannot completely prevent OHSS.

VEGF serum levels are associated with the likelihood of developing OHSS and with clinical feature [49, 50]. After stimulated by HCG, granulosa-lutein cells produce and release high level of VEGF, which interact with VEGF receptor in the endothelial cells membrane. A study by He et al. found that Letrozole significantly decreases the VEGF level, and the VEGF level and the incidence of moderate and severe OHSS decreased with the increase dose of Letrozole [31]. Mai et al.’s study found that the VEGF level was significantly higher in Letrozole group compared with the aspirin group on HCG plus 7 days [30]. Regrettably, not all studies included in our meta-analysis have detected the expression of VEGF. The association between Letrozole administration and the level of VEGF still need further study.

A strength of systematic reviews is the more precision estimate with pooled ORs than the individual studies. The combined estimate indicated that Letrozole administration could decrease the incidence of total OHSS and moderate + severe OHSS, however have no effect on prevention of mild, moderate, and severe OHSS, individually. In addition, there were also some limitations. A major drawback of the present systematic review was the significant heterogeneity among these included studies’ characteristics: different study type (Prospective / Retrospective), different treatment type (IVF/ICSI/PGD), different dose of Letrozole administration (2.5 mg/5.0 mg/7.5 mg), and different treatments in control group (Aspirin/HES/Ca/Placebo). Besides, small sample size of study and lack of adjustment for meaningful confounders were the limitations of the present review. Although there were many drawbacks, the present systematic review and meta-analysis come into a valuable summary of the results of scientific publication so far.

## Conclusion

The findings of this systematic review demonstrated that Letrozole administration after oocyte retrieval in high-risk women has no beneficial effect on the prevention of mild, moderate, and severe OHSS, individually; whereas could decrease the total incidence of moderate + severe OHSS. As the number of events in some studies is relatively small and the characteristics of studies are diversity, Letrozole should not be considered as the first-line treatment for prevention of OHSS. Further cohort studies are needed to explore the effect of administration on the prevention of OHSS.

## Supplementary information

**Additional file 1: Figure S1.** Funnel plot of analysis for the effect of letrozole administration on prevention of total early OHSS, showing the results of Eggers to assess publication bias.

**Additional file 2: Figure S2.** Funnel plot of analysis for the effect of letrozole administration on prevention of mild early OHSS, showing the results of Eggers to assess publication bias.

**Additional file 3: Figure S3.** Funnel plot of analysis for the effect of letrozole administration on prevention of severe early OHSS, showing the results of Eggers to assess publication bias.

**Additional file 4: Figure S4.** Funnel plot of analysis for the effect of letrozole administration on prevention of moderate + severe early OHSS, showing the results of Eggers to assess publication bias.

**Additional file 5: Figure S5.** Funnel plot of analysis for the effect of letrozole administration on prevention of moderate early OHSS, showing the results of Eggers to assess publication bias.

## Data Availability

The datasets supporting the conclusions of this article are included within the article and its additional file.

## Abbreviations

ART: Assisted reproductive technology
CI: Confidence interval
ICSI: Intracytoplasmic sperm injection
OHSS: Ovary hyperstimulation syndrome
OR: Odds ratio
